# A Perspective on the Role of Dynamic Alternative RNA Splicing in the Development, Specification, and Function of Axon Initial Segment

**DOI:** 10.3389/fnmol.2019.00295

**Published:** 2019-12-05

**Authors:** Takatoshi Iijima, Takeshi Yoshimura

**Affiliations:** ^1^Department of Molecular Life Science, Division of Basic Medical Science and Molecular Medicine, School of Medicine, Tokai University, Isehara, Japan; ^2^Department of Child Development and Molecular Brain Science, United Graduate School of Child Development, Osaka University, Suita, Japan

**Keywords:** alternative splicing, axon initial segment, Rbfox, ankyrin, neurofascin, spectrin, neuronal activity, homeostatic plasticity

## Abstract

Alternative splicing is a powerful mechanism for molecular and functional diversification. In neurons, alternative splicing extensively controls various developmental steps as well as the plasticity and remodeling of neuronal activity in the adult brain. The axon initial segment (AIS) is the specialized compartment of proximal axons that initiates action potential (AP). At the AIS, the ion channels and cell adhesion molecules (CAMs) required for AP initiation are densely clustered *via* the scaffolding and cytoskeletal proteins. Notably, recent studies have elucidated that multiple AIS proteins are controlled by extensive alternative splicing in developing and adult brains. Here, we argue the potential role of dynamic regulation of alternative splicing in the development, specification, and functions of the AIS. In particular, we propose the novel concept that alternative splicing potentially modulates the structural and functional plasticity at the AIS.

## Introduction

Alternative RNA splicing through the selective exclusion or inclusion of pre-mRNA sequences is a powerful system for transcriptomic diversity. Alternative splicing decisions are dynamic during neural development (Kalsotra and Cooper, 2011; Vuong et al., 2016), which serves as a developmental switch to facilitate neural differentiation and synapse maturation. For example, developmental shift in alternative splicing of neurexin, a synaptogenic receptor, is crucial for synapse assembly in the cerebellum (Iijima et al., 2011). The expression of PSD-95 is controlled *via* developmentally regulated alternative splicing by the polypyrimidine tract binding proteins (PTBPs) whose sequential downregulation is necessary for glutamatergic synapse maturation (Zheng et al., 2012).

This could be the case with axon development and specification. The axon initial segment (AIS) is a specialized compartment of proximal axons. AIS is a key structure that maintains neuronal polarity and exerts neuronal excitability to initiate action potentials (APs) within neurons (Debanne et al., 2011). AIS functions depend on the local enrichment of a macromolecular complex composed of voltage-gated ion channels, the cell adhesion molecules (CAMs) *via* a submembranous scaffold of ankyrinG (ankG), and βIV spectrin. Notably, these AIS proteins have several alternatively spliced isoforms that are dynamic (Hassel et al., 1997; Suzuki et al., 2017; Jacko et al., 2018; Ogawa et al., 2018). Additionally, alternative splicing shows distinct patterns in a regional/cell type-specific manner (Li et al., 2007; Iijima et al., 2016). Several studies on the biochemical and physiological characteristics of AIS reveal remarkable diversity in AIS structure and function between the neuronal cell types (Rasband, 2010; Bender and Trussell, 2012; Kole and Stuart, 2012; Leterrier and Dargent, 2014). Our recent study found spatial difference in alternatively spliced isoforms of AIS proteins (Suzuki et al., 2017), which suggests that alternative splicing may shape the identity of AIS between neuronal cell types. Considering this point of view, we first discuss the involvement of the molecular repertories generated by alternative splicing in the development and diversity at the AIS.

Furthermore, neuronal activity modulates alternative splicing of multiple neural genes *via* specific signaling pathways (Razanau and Xie, 2013). This could play a critical role in homeostatic plasticity-dependent changes at the synapses (Mu et al., 2003; Iijima et al., 2011; Ding et al., 2017). Similarly, AIS also undergoes plasticity-dependent changes in structure and function due to age, disease, and homeostatic neuronal activity (Grubb et al., 2011; Yoshimura and Rasband, 2014; Yamada and Kuba, 2016; Huang and Rasband, 2018). Our recent finding has revealed that neuronal activity-dependent shift in alternatively spliced isoforms at the AIS (Suzuki et al., 2017) is possibly linked to AIS plasticity. In this perspective review, we argue the potential role of neuronal alternative splicing in plasticity-dependent regulation at the AIS.

## Temporal Changes in Alternatively Spliced Isoforms of AIS Proteins During Development

### Neurofascin (NF)

NF is a polymorphic cell surface protein that has approximately 50 isoforms due to extensive alternative splicing of the NF gene (*Nfasc*) during neural development (Hassel et al., 1997). Alternative splicing of segments on the proximal ectodomain is particularly crucial for generating the four major NF isoforms in the mammalian central nervous system (CNS; Kriebel et al., 2012). Three of these isoforms, NF186, NF180, and NF140 (NF166 in chicken), are neural isoforms, and the fourth is found in glia (Kriebel et al., 2012). NF consists of a set of six Ig-like domains that are common to all of its isoforms and up to five variable FNIII-like domains. The four NF isoforms differ in their combination of FNIII-like domains, as well as the presence of a PAT domain. Alternatively, spliced events provide most of the structural diversity of the NF ectodomain. The three neural NFs demonstrate distinct functions in developing and adult brains. For example, the major neural isoform, NF186, predominantly confers stabilization to AIS and the nodes of Ranvier in adults (Zonta et al., 2008). The other neural isoforms, NF140 and NF180, are embryonic protein variants that regulate neurite outgrowth (Volkmer et al., 1996; Ratcliffe et al., 2001; Pruss et al., 2006; Zhang et al., 2015). In accordance with their distinct function, the embryonic isoforms are largely converted to the adult NF186 isoform during neural development and differentiation (Hassel et al., 1997; Suzuki et al., 2017). This process occurs through the inclusion of four tandem exons (i.e., exons 26, 27, 28, and 29; ex26-29), which encode the fifth FNIII domain and the PAT domain, suggesting the significant role of alternative splicing shift in neuronal polarity and axon development.

### AnkyrinG (AnkG) and βIV Spectrin

AnkG and βIV spectrin are characteristic components of the cytoskeleton at the AIS in neurons (Kordeli et al., 1995; Berghs et al., 2000). βIV spectrin plays a crucial role in linking the ankG/Na^+^ channel membrane protein complex to the actin cytoskeleton (Yang et al., 2007; Ho et al., 2014). Deficiency of ankG and βIV spectrin disrupts AIS assembly and function (Zhou et al., 1998; Komada and Soriano, 2002). Thus, both ankG and βIV spectrin are essential for proper clustering of ion channels at the AIS and the axon domain organization. AnkG has multiple alternatively spliced isoforms, which are developmentally altered. In the CNS, the 190-, 270-, and 480-kDa ankG are found: the 190-kDa isoform is abundant in unmyelinated axons, 270- and 480-kDa isoforms are localized in the AISs and the nodes of Ranvier of myelinated axons (Zhang and Bennett, 1998; Rubtsov and Lopina, 2000; Bennett and Lorenzo, 2013). Recent studies have shown that 33-nt of a cassete exon (exon 34) in the ankG gene (*Ank3*), which encodes a small peptide just upstream of the ZU5 domain and which changes the affinity of spectrin binding, is skipped during the early developmental period (Jacko et al., 2018; Ogawa et al., 2018). βIV spectrin has six splice variants (βIVΣ1–βIVΣ6; Berghs et al., 2000; Komada and Soriano, 2002). Two variants, βIVΣ1 and βIVΣ6, are thought to be at the AIS and nodes of Ranvier (Komada and Soriano, 2002; Lacas-Gervais et al., 2004). A recent study showed that the predominant neuronal βIV spectrin splice variant detected in the developing brain switches from βIVΣ1 to βIVΣ6 (Yoshimura et al., 2017). The shorter of the two isoforms, βIVΣ6, has a large deletion of the N-terminus containing actin-binding domain and several spectrin repeats. The expression levels of ankG splice variants seem to change in keeping with βIV spectrin splice variant switch. Thus, these studies suggest that alternative splicing is a developmental switch for proper AIS formation and organization *via* submembranous scaffold in addition to extracellular proteins.

## Spatial Difference in Alternatively Spliced Isoforms of AIS Proteins Between Brain Regions/Cell Types

The expression and distribution of voltage-gated Na^+^ and K^+^ channels at the AIS vary among neuronal cell types (Lorincz and Nusser, 2008). In the cortical pyramidal neurons, Na_V_1.2 and Na_V_1.6 are enriched in the proximal and distal regions of the AIS, respectively (Hu et al., 2009). Thus, it has been known that AIS constituents are highly different between neuronal cell types (Rasband, 2010; Bender and Trussell, 2012; Kole and Stuart, 2012; Leterrier and Dargent, 2014); different classes of neurons have distinct types, distributions, and/or combinations of voltage-gated Na^+^, K^+^, and Ca^2+^ channels (Na_V_, K_V_, and Ca_V_) at the AIS. Therefore, difference in the components of these ion channels localized at the AIS is likely to contribute to the diversity of firing properties.

In addition to different components of ion channels, our recent study revealed that alternatively spliced isoforms at the AIS are different between neuronal cell types. As described above, the alternative splicing of the NF gene (*Nfasc*) is developmentally regulated; the embryonic isoforms are largely converted to the adult NF186 isoform during neural development and differentiation (Hassel et al., 1997). Unexpectedly, we recently found that the isoform patterns are distinct between forebrains and hindbrains (Suzuki et al., 2017); the ratio of NF186/NF140 is significantly lower in the hindbrain regions, especially the cerebellum, due to the lower inclusion of exons 26–28 encoding FNIII-like domains and a PAT domain during *Nfasc* splicing. We further revealed that the *Nfasc* splicing is different at the cell-type level in the cerebellum, with *Nfasc186* being expressed in Purkinje cells and exclusively absent from cerebellar granule cells (CGCs; Suzuki et al., 2017), suggesting that alternative *Nfasc* splicing is spatially controlled during cell type resolution in the mouse brain. Such cell type-specific splicing regulation may extend the diversity and complexity of the AIS function in the CNS.

## Neuronal Activity-Dependent Regulation of Alternatively Spliced Isoforms at the AIS: The Implication of AIS Plasticity

AIS is not stable; it undergoes a plasticity-dependent change in structure and function based on the neuronal activity (Grubb and Burrone, 2010; Kuba et al., 2010). AIS plasticity is thought to tune overall neuronal excitability in a homeostatic-like manner. However, many aspects remain to be determined regarding the regulatory mechanism and function underlying the AIS plasticity in the CNS.

**Figure 1:**
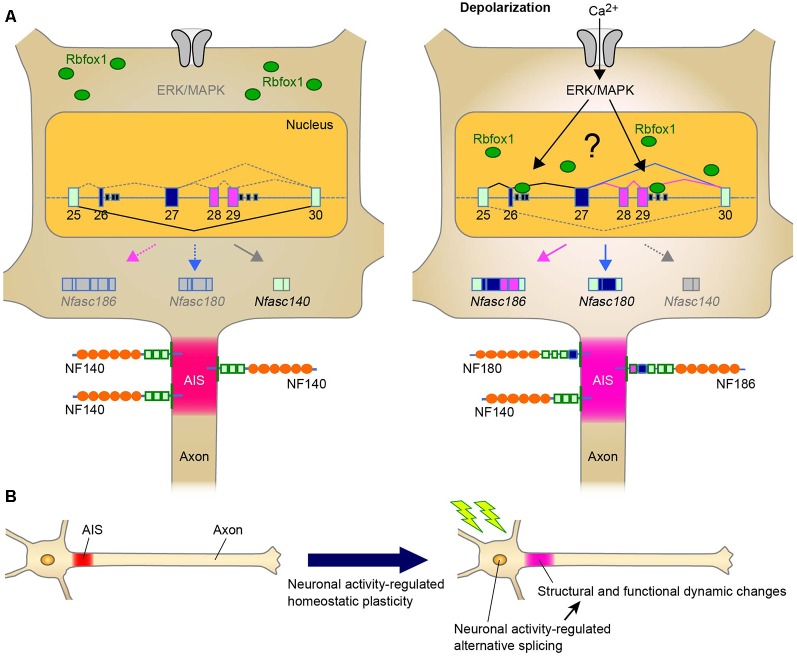
A Schematic representation illustrating the neuronal activity-regulated alternative splicing and homeostatic plasticity at the axon initial segment (AIS). **(A)** Model of activity-dependent molecular change by Rbfox1-mediated alternative splicing during AIS plasticity. Neuronal activity causes the nuclear accumulation of Rbfox1 and induces the inclusion of exons 26–29 at *Nfasc* through extracellular signal-regulated kinase/mitogen-activated protein kinase (ERK/MAPK) signaling. This could result in the replacement of NF140 with NF186 at the AIS. **(B)** Neuronal activity may modulate alternative splicing of genes for AIS proteins. Alternative splicing could contribute to AIS plasticity through changes in the structure and function.

In addition to the role of alternative splicing in AIS assembly during development, another intriguing point would be whether alternative splicing modulates the homeostatic AIS plasticity, and our recent findings strongly support this possibility. We recently found that Rbfox1 regulates neuronal activity-dependent alternative splicing of *Nfasc* in the primary cerebellar neurons (Suzuki et al., 2017; Table 1). The major finding of this study is that, although* Nfasc186* is absent from mature CGCs, Rbfox1 induces a shift in splicing from *Nfasc140* to *Nfasc186* (Figure 1A). Thus, our recent study has suggested that the shift in NF isoform could cause functional changes in the adult stage as well as during the developmental stage.

**Table 1:** List of Rbfox-targeted genes encoding representative axon initial segment (AIS) proteins.

Cellular functions	Proteins (genes)	Altered exons (exon type)	Activity dependency	References
Cell adhesion	Neurofascin (*Nfasc*)	Exon 26–29 (cassete)	Yes (primary neurons)	Suzuki et al. (2017) and Jacko et al. (2018)
Scaffolding	AnkyrinB (*Ank2*)	Exon 46 (cassete)	Non-informative	Jacko et al. (2018)
	AnkyrinG (*Ank3*)	Exon 34	Yes (P19 cells)	Lee et al. (2009) and Jacko et al. (2018)
Ion channel	Na_v_1.6 (*Scn8a*)	Exon 5 (cassete) Exon18 (mutually exclusive)	Non-informative	Gehman et al. (2012), O’brien et al. (2012) and Jacko et al. (2018)
	K_v_7.2 (*Kcnq2*)	Exon 11 (cassete)	Non-informative	Gehman et al. (2012) and Jacko et al. (2018)
	Ca_v_2.2 (*Cacan1b*)	Exon18 (cassete) Exon 25 (cassete)	Yes (Exon 25; P19 cells)	Lee et al. (2009), Allen et al. (2017) and Jacko et al. (2018)

*The table summarizes the genes that are alternatively spliced by Rbfox proteins. Non-informative*.

Chronic changes in neuronal activity induce structural and functional plasticity in specific types of neurons (Grubb and Burrone, 2010; Kuba et al., 2010). Given that Rbfox proteins target multiple genes encoding AIS proteins (Jacko et al., 2018), the neuronal activity-regulated alternative splicing by Rbfox1 proteins may cause several molecular changes during AIS plasticity. This notion could be supported by a previous study that reported that Rbfox1 induces depolarization-dependent splicing shifts in exons at several genes, including *Ank3*, *Cacna1b*, and *Kcnq2*, in a differentiated P19 cell line (Lee et al., 2009; Table 1). Therefore, we speculate that neuronal activity-dependent splicing regulation modulates the plasticity-dependent change at the AIS in the CNS (Figure 1B). Notably, we recently observed that in addition to the shift in *Nfasc* splicing, depolarization dynamically changed the structure and function of the AIS in CGCs (Suzuki et al., submitting the manuscript), implying a strong link between activity-regulated alternative splicing and cerebellar AIS plasticity. Although future studies are necessary to obtain the direct evidence of the control of AIS plasticity by alternative splicing, our recent findings in cerebellar neurons could add to the further understanding of the molecular mechanism underlying axon dynamics.

## The Regulatory Mechanism Underlying the Spatiotemporal Control of AIS by Alternative Splicing

As described above, alternatively spliced isoforms of several AIS proteins are dynamically altered during neural development, implying that change in splicing isoform could be required for proper AIS formation. Indeed, RNA-binding protein Rbfox-mediated splicing was recently reported to promote AIS assembly (Jacko et al., 2018). Rbfox proteins (Rbfox1/2/3) are a family of tissue-specific splicing regulators (Kuroyanagi, 2009). Jacko et al. (2018) generated an Rbfox1/2/3 triple knockout (TKO) embryonic stem cell (ESC) line to overcome the functional redundancy of three Rbfox proteins and found that neurons differentiated from Rbfox TKO ESCs had impaired AIS assembly. Strikingly, Rbfox proteins target approximately 50% of the genes encoding AIS proteins (i.e., ankG, NF, Na_V_, K_V_, and Ca_V_; Table 1). Indeed, several previous studies have reported that Rbfox proteins regulate the alternative splicing of *Scn8a*, *Cacna1b*, and *Kcnq2* genes that encode Na_V_1.6, Ca_V_2.2, and Ka_V_7.2, respectively, all of which are enriched at the AIS (Gehman et al., 2012; O’brien et al., 2012; Allen et al., 2017). Mice with conditional knockout of Rbfox1 and Rbfox2 in mature Purkinje cells exhibited highly irregular neuronal firing, a hallmark of functional AIS impairment (Gehman et al., 2012). Particularly, Jacko et al. (2018) revealed that the developmental switch in *Ank3* splicing was the most critical for AIS assembly. The skipping of exon 34, which is located upstream to the ZU5 domain and enables the interaction of ankG with βIV spectrin, was markedly impaired in Rbfox TKO neurons and could be dominantly responsible for the severe perturbation of AIS.

In addition, the alternative splicing of *Ank3* exon 34 may be regulated in a neuronal region or cell type-specific manner. Elavl3 is an RNA-binding protein that is highly expressed in cerebellar Purkinje cells. The skipping of exon 34 is misregulated in the cerebellum of Elavl3 KO mice (Ogawa et al., 2018). The length of the AIS is shortened in the Purkinje cells of adult Elavl3 KO mice, suggesting a significant role of Elavl3-mediated splicing in AIS formation and maintenance.

Furthermore, as mentioned in “Neuronal Activity-Dependent Regulation of Alternatively Spliced Isoforms at the AIS: The Implication of AIS Plasticity” section, we recently found that Rbfox1 is a key protein in the activity-regulated selection of the *Nfasc* isoform that specifically includes the exons 26−29 in the primary neurons (Suzuki et al., 2017; Figure 1A). This activity-dependent effect is specific to Rbfox1 over the other family proteins, this is mediated *via* the ERK/MAPK pathway upon high K^+^-induced depolarization. Thus, the discovery of these splicing factors provides further understanding of the dynamic control of the formation and function of the AIS by alternative splicing.

## Open Questions

Increasing evidence indicates that alternative splicing could be crucial for the development, specification, and function of AIS. Notably, Rbfox proteins regulate AIS assembly *via* alternative splicing of multiple genes that encode AIS-related proteins. Particularly, the developmental skipping of exon 34 at *Ank3* by Rbfox proteins likely plays a crucial role in proper AIS formation (Jacko et al., 2018). However, the functional significance of developmental and regional/cell type-specific alternative splicing remains to be identified. For example, there is a splicing shift in the isoforms of βIV spectrin and NF during development, but the functional differences between the embryonic and adult isoforms of these AIS proteins remain elusive.

Despite a developmental shift from full-length βIV spectrin Σ1 into a shorter isoform βIVΣ6, which lacks the actin-binding domain, the spacing of spectrin tetramers between the actin rings is conserved (Yoshimura et al., 2017). It is possible that βIVΣ1 forms a ladder-like lattice structure, and βIVΣ6 makes AIS ultrastructurally more complex in adults. Additional physiological studies in the future with gene manipulation could be necessary for addressing the functional consequence of this conversion.

Although several studies have assessed different usages of NF in the neuronal function between embryonic and adult phases (Kriebel et al., 2012), the functional difference between embryonic and adult isoforms remains unclear. Expression of each isoform (NF140 and NF186) in *Nfasc*-KO background showed no distinct subcellular localizations and function of these isoforms at the AIS and nodes of Ranvier in mice (Zhang et al., 2015); thus, loss of adult isoforms could be compensated at the same level by expression of the embryonic isoform in adults. However, because NF140 remains expressed in some regions of the adult mouse brain, an embryonic isoform could exert some of the similar biological activities even in the mature CNS. What, therefore, is the functional difference of NF isoforms among different neuronal cell types? Some aspects of developmental and regional/cell type splicing on biological significance still remain controversial and, thus, should be addressed in future studies.

## Future Directions

Herein, the most novel perspective would be the potential role of neuronal alternative splicing in plasticity-dependent regulation at the AIS (Figure 1B). As described above, several studies have revealed that multiple genes that encode AIS-related proteins are alternatively spliced. However, it is not yet clear how many of these genes are regulated by neuronal activity. It is likely that Rbfox1 is one of the key splicing regulators driving the molecular changes during AIS plasticity. Therefore, it would be interesting to uncover the core mechanisms and functional aspects underlying the activity-dependent splicing programming by Rbfox1.

Furthermore, another intriguing point is the implication of aberrant alternative splicing at the AIS in the development of several neurological disorders. *ANK3* has been associated with several psychiatric disorders, including schizophrenia, bipolar disorder, and autism spectrum disorder (Huang and Rasband, 2018). AnkG undergoes extensive alternative splicing, and ankG splice variants have been proposed to contribute to bipolar disorder and epilepsy (Lopez et al., 2017). *SPTBN4* (gene encoding βIV spectrin) has also been associated with intellectual disability, congenital hypotonia, and motor axonal neuropathy (Wang et al., 2018). NF is a target for autoantibody-mediated axonal injury (Mathey et al., 2007). Thus, it is possible that splicing abnormalities of genes encoding AIS proteins may cause these psychiatric and neurological disorders. Therefore, further studies may provide a profound understanding of the pathophysiology of and novel therapeutic strategy for these neurological disorders.

## Data Availability Statement

The datasets generated for this study are available on request to the corresponding author.

## Author Contributions

TI and TY wrote the manuscript.

## Conflict of Interest

The authors declare that the research was conducted in the absence of any commercial or financial relationships that could be construed as a potential conflict of interest.
